# The hypertriglyceridemic-waist phenotype as a valuable and integrative mirror of metabolic syndrome traits

**DOI:** 10.1038/s41598-021-01343-x

**Published:** 2021-11-08

**Authors:** Begoña de Cuevillas, Ismael Alvarez-Alvarez, Jose I. Riezu-Boj, Santiago Navas-Carretero, J. Alfredo Martinez

**Affiliations:** 1 Department of Nutrition, Food Sciences and Physiology, Center for Nutrition Research, University of Navarra, Calle Irunlarrea 1, 31008 Pamplona, Spain; 2 IdisNA Health Research Institute of Navarra, Pamplona, Spain; 3 CIBER Physiopathology of Obesity and Nutrition (CIBEROBN), Institute of Health Carlos III, 28029 Madrid, Spain; 4 Precision Nutrition Program, Cardiometabolic IMDEA Food, 28049 Madrid, Spain

**Keywords:** Endocrine system and metabolic diseases, Dyslipidaemias, Risk factors

## Abstract

Rates of non-communicable diseases (NCDs), such as obesity, diabetes, cardiovascular events and cancer, continue to rise worldwide, which require objective instruments for preventive and management actions. Diverse anthropometric and biochemical markers have been used to qualitatively evaluate degrees of disease, metabolic traits and evolution of nutritional status. The aim of this study was to integrate and assess the interactions between an anthropometric measurement, such as waist circumference (WC), and biochemical data, such as the triglyceride glucose index (TyG), in order to individually characterize metabolic syndrome (MetS) features considering the hypertriglyceridemic waist phenotype as a marker. An ancillary cross-sectional study was conducted using anthropometric measurements, such as weight, height, waist and hip circumferences, as well as fasting biochemical data of 314 participants. Different indices based on WC (WC, WC*TG and WC*TyG) were estimated to compute MetS components and accompanying comorbidities. ROC curves were fitted to define the strength of the analyses and the validity of the relationships. Associations were confirmed between anthropometric, biochemical and combined indices with some chronic disease manifestations, including hyperglycemia, hypertension and dyslipidemia. Both WC*TG and WC*TyG indices showed similar performance in diagnosing MetS (area under the ROC curve = 0.81). Interestingly, when participants were categorized according to a reference value of the WC*TyG index (842.7 cm*mg/dl), our results evidenced that subjects classified over this limit presented statistically higher prevalence of MetS and accompanying individual components with clinical relevance for interventions. These results revealed that WC*TyG mirrors the hypertriglyceridemic phenotype, which suggests may serve as a good indicator to define the metabolic syndrome phenotype and a suitable, sensitive, and simple proxy to complement others. A reference point was proposed with a good clinical performance and maximized sensitivity and specificity values.

## Introduction

Noncommunicable diseases (NCDs)—mainly cardiovascular diseases, cancers, chronic respiratory illness and diabetes—are important worldwide causes of death^1^. The prevention of these diseases represents a challenge for health and populations’ wellbeing, given that NCDs are the cause of 71% of world deaths^2,3^. In this context, metabolic syndrome (MetS) is defined as a cluster of 3 or more risk factors, including excessive adiposity, hypertension, dyslipidemia and elevated fasting blood glucose, which is receiving growing clinical and public health attention^4,5^. The clinical need for developing valid and unified diagnosis and interpretation reference criteria is demonstrated by the current existence of diverse definitions provided by the Adult Treatment Panel III (ATP III), the European Group for the Study of Insulin Resistance (EGIR) and the International Diabetes Federation (IDF)^6^.

In this context, anthropometric markers, such as weight, height, skinfolds and circumferences, have been used to evaluate the relationships between disease risk and nutritional status^7^. Thus, previous studies have reported the usefulness of waist circumference (WC), waist-to-hip ratio (WHR) and body mass index (BMI) for predicting metabolic diseases and mortality^8^. Furthermore, the waist-to-height ratio (WHtR) may be an effective screening tool for the evaluation of abdominal adiposity and associations with cardiometabolic adverse features, while measures of visceral fat and related markers are linked with incident MetS, morbidity and mortality prevalence^9,10^.

In addition, biochemical markers are also known as reliable tools to define and discriminate a number of metabolic disorders^11^. For instance, the visceral adiposity index (VAI) and HDL-cholesterol ratio were found to be effective instruments for diabetes and cardiovascular risk prediction^12,13^. Furthermore, high triglycerides (TG) levels were associated with insulin resistance and cardiometabolic events^14,15^. In this context, the triglyceride glucose index (TyG) and the combined index WC^*^TyG have been proposed as suitable surrogate markers of insulin resistance and inflammation as well as accompanying metabolic disturbances affecting liver, kidney and heart pathological conditions^16–18^.

Previous studies have assessed the associations of some of the aforementioned indicators with the prevalence of type 2 diabetes, coronary heart disease and liver disease, however there is a lack of studies concerning the definition of reference values based on combined anthropometric and biochemical indices, where excessive adiposity and lipid/glucose metabolism impairments coexist^19–22^. Indeed, a relation between fatty acids and waist circumference with metabolic health have been established^23^.

Therefore, the current study was designed to evaluate the possible associations between various anthropometric and biochemical parameters, or combined determinants such as the WC^*^TyG index that reflects the hypertriglyceridemic phenotype of MetS’s accompanying components in order to individually categorize metabolic syndrome and facilitate a personalized management.

## Material and methods

### Study population

The initial population of the current study (n = 314) included subjects between 18–76 years old of which 216 were overweight and obese subjects from the database of the Center for Nutrition Research of the University of Navarra (Spain) that fulfilled all the experimental inclusion/exclusion criteria and ethical requirements. The study protocol was approved by the Research Ethics Committee of the University of Navarra (ref. 132/2015). The research was performed in accordance with the ethical guidelines of the Declaration of Helsinki^24^. All participants provided written informed consent, which was signed and filled after they received a printed information sheet and additional verbal explanation of the protocol. The sample size was defined by the data availability, which is generally accepted in clinical scenarios, although type I and type II errors cannot be discarded.

Inclusion criteria were as follows: to be over 18 years old, with BMI over 25 kg/m^2^, and not medicated for any of the following diseases: hypertension, hypertriglyceridemia or dyslipidemia, type 2 diabetes. Exclusion criteria, in addition to suffering from any of the previously mentioned conditions, included being normal-weight/underweight or suffer from any other metabolic condition, such as thyroid diseases, type 1 diabetes or type of cancer.

### Study design

This ancillary research was a cross-sectional analysis of data from the Center for Nutrition Research’s database. In this study’s particular case, a sample size was not calculated, and all subjects complying with the inclusion criteria were included. The field work was carried out by trained staff from the Center for Nutrition Research, including nurses, dietitians and physicians following the required ethical criteria.

### Measurements

Anthropometric indices were measured in fasting conditions in the morning, with the subjects wearing underwear, by a trained dietitian following validated procedures^25^. Body weight (kg) was assessed using a calibrated scale and height (cm) was measured using a wall-mounted stadiometer, while body composition was determined by bioimpedance (TANITA BC-418 Scale, Tokyo, Japan) following standardized protocols. BMI was calculated as the ratio between weight and squared height (kg/m^2^). Waist circumference (cm) was evaluated both with the participants standing and with them lying down, while hip circumference (cm) was measured standing, using a non-elastic measure tape as described elsewhere^26^. This measurement method used has shown in other studies to have good validity and excellent reliability^26^. Body fat mass was analyzed by dual energy x-ray absorptiometry (DEXA) scan (DEXA Lunar Prodigy 6.0, GE Medical Systems, Madison, WI, USA) following a standardized protocol. Systolic blood pressure (SBP, mmHg) and diastolic blood pressure (DBP, mmHg) were measured in duplicate with an automated sphygmomanometer, according to the standardized criteria described by the World Health Organization and the International Society of Hypertension^27^.

Venous blood samples were drawn from each participant, in the morning, by venipuncture after a 12-h overnight fast. Fasting plasma glucose (FPG), total cholesterol, HDL-c, and TG were determined in an automatic analyzer Pentra C200 (HORIBA Medical, Madrid, Spain) with appropriate commercial kits provided by the company. LDL-cholesterol was calculated using the Friedewald equation (LDL-c = total cholesterol − HDL-c − TG/5), as described elsewhere^28^.

From the anthropometric and biochemical data, the following indices were computed: TyG (ln [TG (mg/dl) ^*^ FPG (mg/dl)/2]), the WC^*^TG index (WC (cm) ^*^ TG (mg/dl)) and WC^*^TyG (WC (cm) ^*^ TyG) as described elsewhere^20,29^. The VAI index was calculated according to the formula proposed by Amato et al*.*^13^: Men: [WC (cm)/(39.68 + 1.88 ^*^ BMI (kg/m^2^))] ^*^ (TG (mg/dl)/1.03) ^*^ (1.31/HDL-c) (mg/dl); Women: [WC (cm)/(36.58 + 1.89 ^*^ BMI(kg/m^2^))] ^*^ (TG (mg/dl)/0.81) ^*^ (1.52/HDL-c (mg/dl)).

### Outcomes

High blood glucose was defined as having fasting blood glucose ≥ 100 mg/dl. A participant was defined as hypertensive if the average systolic blood pressure was ≥ 130 mmHg or diastolic blood pressure was ≥ 80 mmHg. Dyslipidemia was considered as having either low HDL-c (< 40 mg/dl in men or < 50 mg/dl in women) as previously described^30^ and triglycerides ≥ 150 mg/dl. Abdominal obesity was determined by having ≥ 88 cm in women and ≥ 102 cm in men^31^. MetS was defined as having at least 3 of the following criteria: (1) high fasting blood glucose, (2) hypertension, (3) dyslipidemia, (4) abdominal obesity as stated elsewhere^32^.

### Covariables

Baseline dietary intake was assessed with a previously validated 137-item food frequency questionnaire^33^. Energy intake was further calculated with an ad hoc computed program based on the standard Spanish food composition tables^34^. Adherence to a Mediterranean Diet was calculated with the Mediterranean Diet Score, which ranged 0–9 points^35^. Physical activity (METs/h) was assessed with the Spanish validated version based of the physical activity questionnaire used in the Nurses’ Health Study and the Health Professionals’ Follow-up Study^36^.

### Statistical analysis

A descriptive analysis of baseline characteristics across sex-specific and age-specific (below and above the median age) groups was performed. For categorical variables chi squared test for independence was used. Continuous data were computed as means and standard deviations, and differences were tested with the Student’s t test.

Receiver operating characteristics (ROC) curves were fitted to define the relative diagnostic strength of the above-mentioned anthropometric indices for the correct diagnosis of high blood glucose, hypertension, dyslipidemia and specifically MetS. We used the area under the curve (AUC) to quantify the accuracy. We interpreted an AUC between 0.90–0.80 as a good diagnostic test, and between 0.80–0.70 and 0.70–0.60 as fair and poor diagnostic tests, respectively. The optimal reference point was calculated using the Youden index^37^ for choosing the threshold value where values of both sensitivity and specificity are maximized.

Interactions between the anthropometric indices and sex and age were tested by fitting models without and with the interaction product term and using the likelihood ratio test. In addition, we performed stratified analyses according to the results.

We fitted adjusted linear regression models to study the associations between anthropometric indices (BMI, WC, WC^*^TG and WC^*^TyG) and metabolic syndrome and its components. Additionally, we fitted multivariable adjusted logistic regression models to study the associations between being classified above a previously calculated cut-off point and the risk of presenting high blood glucose, hypertension, dyslipidemia and MetS. All models were adjusted for potential confounders (age, sex, physical activity, energy intake, adherence to a Mediterranean dietary pattern, family history of obesity, and education level). All p values were two-tailed, and a p value lower than 0.05 was deemed statistically significant. Analyses were performed using Stata version 13.0 (StataCorp, College Station, TX, USA).

## Results

Baseline characteristics by sex of the analyzed sample are reported (Table 1). Compared to men, women showed lower prevalence of prediabetes, but not diabetes, (19.9% vs 31.5%; p = 0.028), high blood glucose (21.3% vs 35.9%; p = 0.008), and MetS (18.4% vs 34.5%; p = 0.001). In addition, women had lower scores for several indices (WC standing (94.6 ± 14.1 vs 104.0 ± 13.0; p < 0.001) and lie down (94.7 ± 8.1 vs 104.3 ± 9.1; p < 0.001), Waist-to-Hip Ratio (86.1 ± 8.5 vs 97.9 ± 8.4; p < 0.001), WC^*^TG (107.1 ± 55.4 vs 155.1 ± 93.2; p < 0.001) and WC^*^TyG (828.7 ± 112.9 vs 934.7 ± 106.4; p < 0.001)). The female group also evidenced lower values for weight (78.7 ± 14.5 vs 93.2 ± 14.6; p < 0.001) and glucose (94.9 ± 14.5 vs 100.4 ± 12.4; p = 0.002), meanwhile te opposite was found for fat mass (34.9 ± 7.7 vs 28.8 ± 6.7; p < 0.001) and HDL-cholesterol rates (59.0 ± 12.8 vs 46.9 ± 8.4; p < 0.001) as found (Table 1).

**Table 1:** Baseline characteristics of the study sample.

	Men	Women	p-value	< 46 years	≥ 46 years	p-value
Subjects (n)	95	219		149	165	
Age (years)	45.9 ± 9.7	45.5 ± 10.9	0.744	36.8 ± 6.7	53.6 ± 6.1	** < *0.001***
**Sex (%)**						0.472
Men	100	–		32.2	28.5	
Women	–	100		67.8	71.5	
Weight (kg)	93.2 ± 14.6	78.7 ± 14.5	** < *0.001***	88.7 ± 13.1	86.8 ± 12.8	0.219
BMI (kg/m^2^)	30.3 ± 4.5	29.8 ± 5.1	0.747	31.1 ± 3.2	32.1 ± 3.6	***0.018***
VAI (AU)^§^	99.7 ± 69.2	90.4 ± 61.6	0.242	83.4 ± 55.2	102.0 ± 70.0	***0.011***
WC standing (cm)	104.0 ± 13.0	94.6 ± 14.1	** < *0.001***	99.9 ± 10.3	104.2 ± 10.4	** < *0.001***
WC lie down (cm)	104.3 ± 9.1	94.7 ± 8.1	** < *0.001***	95.4 ± 9.2	99.1 ± 9.4	***0.034***
Waist-to-hip ratio (cm)	97.9 ± 8.4	86.1 ± 8.5	** < *0.001***	89.1 ± 8.8	94.1 ± 9.2	** < *0.001***
Waist-to-height ratio (cm)	59.4 ± 7.6	58.3 ± 8.8	0.251	59.3 ± 5.5	63.5 ± 6.2	** < *0.001***
Bioimpedance fat mass (kg)	28.8 ± 6.7	34.9 ± 7.7	** < *0.001***	32.6 ± 8.6	33.5 ± 7.3	0.302
Glucose (md/dl)	100.4 ± 12.4	94.9 ± 14.5	***0.002***	92.6 ± 7.8	100.1 ± 17.2	** < *0.001***
Total cholesterol (mg/dl)	219.3 ± 39.0	214.6 ± 38.0	0.321	205.3 ± 36.3	225.6 ± 37.5	** < *0.001***
HDL cholesterol (mg/dl)	46.9 ± 8.4	59.0 ± 12.8	** < *0.001***	53.8 ± 12.7	56.6 ± 13.0	0.051
Triglycerides (mg/dl)	125.7 ± 73.4	93.9 ± 43.9	** < *0.001***	92.4 ± 46.1	113.6 ± 62.6	***0.001***
TyG (mg/dl)	8.6 ± 0.5	8.3 ± 0.5	** < *0.001***	8.3 ± 0.5	8.5 ± 0.5	** < *0.001***
WC*TyG (cm*mg/dl)	934.7 ± 106.4	828.7 ± 112.9	** < *0.001***	826.5 ± 108.5	891.6 ± 123.7	** < *0.001***
WC*TG (cm*mg/dl)	155.1 ± 93.2	107.1 ± 55.4	** < *0.001***	105.6 ± 57.1	136.1 ± 81.2	** < *0.001***
Hypertension (%)	73.5	43.1	** < *0.001***	46.3	71.5	** < *0.001***
SBP (mm Hg)	134.3 ± 17.0	120.9 ± 16.2	** < *0.001***	123.4 ± 15.8	132.0 ± 17.2	** < *0.001***
DBP (mm Hg)	81.9 ± 11.6	76.0 ± 10.0	** < *0.001***	76.6 ± 10.7	82.0 ± 9.8	** < *0.001***
HOMA-IR	1.77;1.09–2.76	1.48;1.02–2.30	0.153	1.50;098–2.05	1.68;1.06–2.60	***0.050***
CRP	1.39;.822-.46	1.71;1.07–4.59	0.568	1.53;1.02–3.72	1.61;1.09–3.27	*0.698*
Family history of obesity (%)	40.5	49.2	0.249	54.4	46.3	0.154
Diabetes (%)	4.3	1.4	0.119	0	4.4	***0.011***
Prediabetes (%)	31.5	19.9	***0.028***	14.0	31.9	** < *0.001***
High blood glucose (%)	35.9	21.3	***0.008***	14.0	36.3	** < *0.001***
Hypercholesterolemia (%)	70.7	64.0	0.260	55.2	75.6	** < *0.001***
Dyslipidemia (%)	30.0	22.0	0.106	30.1	29.4	0.895
Metabolic Syndrome (%)	34.5	18.4	***0.001***	20.1	35.2	***0.003***
Adherence to a MedDiet (points)^‡^	4.3 ± 1.7	4.2 ± 1.6	0.435	3.9 ± 1.5	4.4 ± 1.6	***0.003***
Physical activity (METs/week)	31.2 ± 24.2	20.5 ± 16.8	** < *0.001***	25.5 ± 22.3	22.3 ± 17.5	0.164
Energy intake (kJ/d)	13,554 ± 4081	12,030 ± 3800	***0.002***	12,411 ± 3968	12,562 ± 3934	0.736

Variables are shown as mean ± SD, as percentage or as median and interquartile range according to their distribution.

^§^AU: Arbitrary Units.

BMI: body mass index; VAI: Visceral Adiposity Index; WC: waist circumference; TG: triglycerides; SBP: Systolic Blood Pressure; DBP: Diastolic Blood Pressure; CRP: C-reactive protein; MedDiet: Mediterranean diet.

^‡^ Adherence to the Mediterranean diet measured with the Mediterranean Diet Score (35).

When the interactions between the anthropometric indices and sex and age were tested, we only found an interaction between the WC^*^TyG index and sex. Thus, when the WC^*^TyG index increased, men showed a slightly higher risk for presenting MetS (OR = 1.02; 95% CI 1.01–1.03) compared to women (OR = 1.01; 95% 1.01–1.01).

When logistic regression models were fitted, measuring indices as continuous variables, only WC^*^TG and WC^*^TyG indexes were statistically and positively associated with an increased risk for presenting high blood glucose and dyslipidemia. A 1-unit increase in all indices mentioned above was consistently associated with an increased risk of hypertension and MetS (Table 2).

**Table 2:** Associations between indices (measured as continuous variables) and individual metabolic syndrome manifestations.

	High Blood Glucose (n = 294)		Hypertension (n = 294)		Dyslipidaemia (n = 294)		Metabolic syndrome (n = 294)	
	OR (95% CI)	p-value	OR (95% CI)	p-value	OR (95% CI)	p-value	OR (95% CI)	p-value
BMI (kg/m^2^)	1.05 (0.97, 1.14)	0.228	1.24 (1.13, 1.35)	** < *0.001***	1.12 (1.04, 1.21)	***0.025***	1.15 (1.06, 1.25)	** < *0.001***
WC (cm)	1.03 (1.00, 1.06)	0.077	1.06 (1.03, 1.09)	** < *0.001***	1.06 (1.03, 1.02)	** < *0.001***	1.07 (1.03, 1.10)	** < *0.001***
WC*TG (cm*mg/dl)	1.01 (1.00, 1.01)	***0.005***	1.01 (1.00, 1.01)	** < *0.001***	1.04 (1.03, 1.05)	** < *0.001***	1.02 (1.02, 1.03)	** < *0.001***
WC*TyG (cm*mg/dl)	1.01 (1.00, 1.01)	** < *0.001***	1.01 (1.00, 1.01)	** < *0.001***	1.01 (1.01, 1.02)	** < *0.001***	1.01 (1.01, 1.01)	** < *0.001***

Adjusted by age, sex, physical activity, total energy intake, adherence to a Mediterranean diet, family history of obesity and education level.

**Figure 1:**
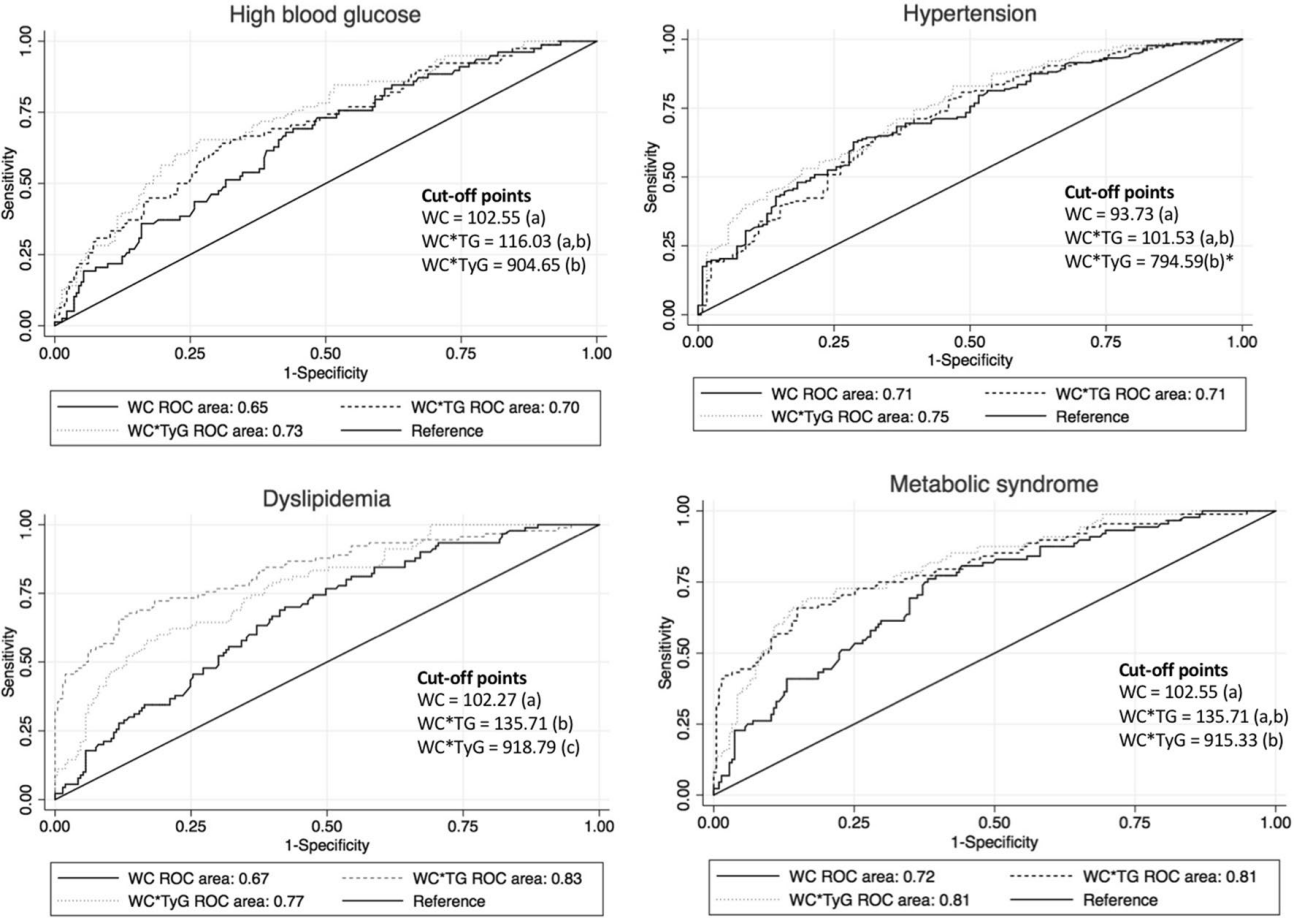
Receiver operating characteristic (ROC) curve analysis for each index (waist circumference, waist circumference-triglycerides, waist circumference-TyG). The different indices were classified in the same group (a, b or c) if we did not find statistical differences between them. Conversely, if statistical differences were noted the indices were classified in different groups. p = 0.063.

ROC analyses were conducted to estimate the diagnosis performance of the analyses (Fig. 1). For either high blood glucose, the largest AUC was observed in WC^*^TyG (0.73), although no statistical differences were found between the WC^*^TG (AUC = 0.70) and WC^*^TyG indices. WC^*^TG was the best index to screen dyslipidemia (AUC = 0.76), compared to both WC and WC^*^TyG (AUC = 0.61 and AUC = 0.69, respectively). No statistical differences between WC^*^TG and both WC and WC^*^TyG referring to dyslipidemia were ascertained. Interestingly, for MetS, WC^*^TyG was classified as a good diagnostic index (AUC = 0.81) by showing significant differences with WC, but not with the WC^*^TG index (Fig. 1). When stratifying by sex, very similar values were observed between men and women with slight differences due to the increase in the sample size and metabolic syndrome showed more substantial differences (men: 0.59, women 0.65, overall 0.72), which should at least be ascribed to the higher sample size analyzed.

Considering specificity and sensitivity parameters, optimal cut-off points were proposed to predict the presence or absence of MetS. These included: 102.27 (sensitivity = 0.68; specificity = 0.73) for WC, 135.71 (sensitivity = 0.73; specificity = 0.70) for WC^*^TG, and 915.33 (sensitivity = 0.74; specificity = 0.72) for WC^*^TyG respectively. Regarding dyslipidemia, the reference values were set to 96.98 for WC, 135.71 for the combined index WC^*^TG, and 918.79 for WC^*^TyG (Fig. 1, Supplemental Table 1).

Participants were categorized according to the specific proposal cut-off points, above or below the reference point (Table 3). Subjects in the higher category of WC^*^TG and WC^*^TyG, compared to those in the lower category, showed over a twofold risk for presenting high blood glucose levels. Those participants with higher WC (OR = 2.88, 95% C.I. = 1.64–5.06), WC^*^TG (OR = 2.82, 95% C.I. = 1.63–4.88) and WC^*^TyG (OR = 3.46, 95% C.I. = 1.91–6.24) showed the highest risks for developing hypertension. Those with higher WC and WC^*^TyG values also had higher risk of dyslipidemia. On the other hand, participants who had higher values of WC (OR = 3.23; 95% C.I. = 1.75–5.93), WC^*^TG (OR = 4.07; 95% C.I. = 2.16–7.66) and WC^*^TyG (OR = 4.60; 95% C.I. = 2.39–8.86) showed the highest risks for developing MetS. Notably, those with higher WC^*^TG had the highest risk for dyslipidemia (OR = 8.82; 95% C.I. = 4.42–17.56) followed by metabolic syndrome features (OR = 4.07; 95% C.I. = 2.16–7.66), high blood glucose (OR = 2.87; 95% C.I. = 1.56–5.26) and hypertension (OR = 2.82; 95% C.I. = 1.63–4.88) (Table 3).

**Table 3:** Associations between indices (below/above this group specific cut-off point) with MetS and recognizable manifestations.

Index	Cut-off	High blood glucose	Cut-off	Hypertension	Cut-off	Dyslipidaemia	Cut-off	MetS
WC	Low (< 102.6)	1 (reference)	Low (< 102.7)	1 (reference)	Low (< 101.2)	1 (reference)	Low (< 102.8)	1 (reference)
	High (≥ 102.6)	1.87 (0.99–3.53)	High (≥ 102.7)	2.88 (1.64–5.06)*	High (≥ 101.2)	3.13 (1.68–5.86)*	High (≥ 102.8)	3.23 (1.75–5.93)*
WC*TG	Low (< 116.0)	1 (reference)	Low (< 101.5)	1 (reference)	Low (< 99.3)	1 (reference)	Low (< 101.5)	1 (reference)
	High (≥ 116.0)	2.87 (1.56–5.26)*	High (≥ 101.5)	2.82 (1.63–4.88)*	High (≥ 99.3)	8.82 (4.42–17.56)*	High (≥ 101.5)	4.07 (2.16–7.66)*
WC*TyG	Low (< 904.7)	1 (reference)	Low (< 794.6)	1 (reference)	Low (< 820.6)	1 (reference)	Low (< 842.7)	1 (reference)
	High (≥ 904.7)	3.76 (1.98–7.14)*	High (≥ 794.6)	3.46 (1.91–6.24)*	High (≥ 820.6)	6.77 (3.32–13.79)*	High (≥ 842.7)	4.60 (2.39–8.86)*

* p < 0.05.

Adjusted for sex, age, physical activity, family history of obesity, energy intake, adherence to a Mediterranean dietary pattern and education level.

The association between indices and diseases stratified by sex evidenced that having higher values of WC (OR = 3.16), WC^*^TG (OR = 3.20) and WC^*^TyG (OR = 4.07) in women is related with higher risk of hypertension, unlike in men. Similarly, a rise in WC^*^TG index was associated with an increased risk of suffering dyslipidemia (OR = 7.07) (Supplemental Table 2).

**Figure 2:**
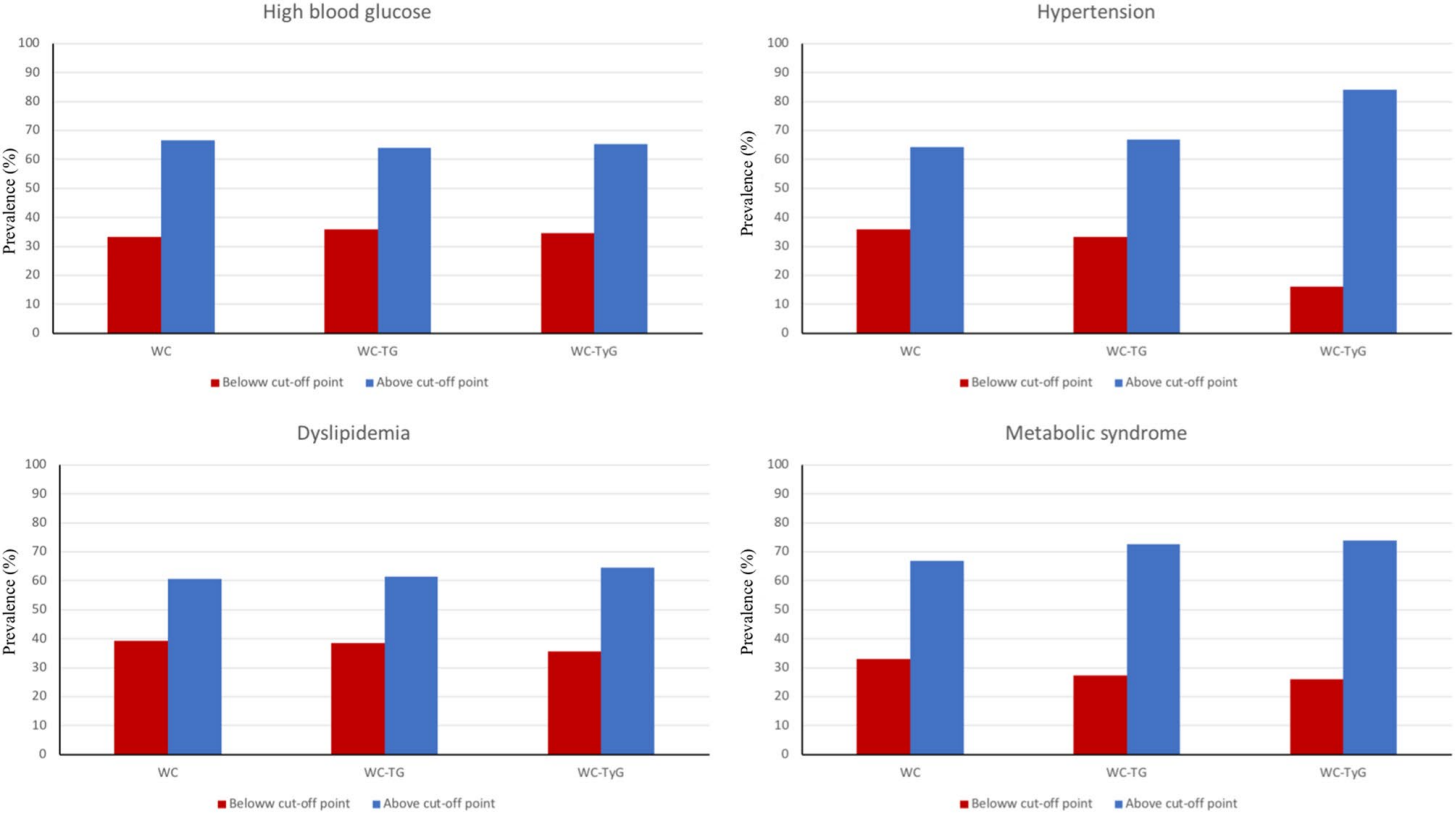
Prevalence of the metabolic syndrome and individual manifestations components according to the designed cut-off points.

After categorizing the participants according to the estimated cut-off point, subjects classified over the cut-off point presented higher prevalence for dyslipidemia, hypertension, high blood glucose and MetS accompanying components. The WC^*^TyG index showed the overall best performance in diagnosing overall MetS and individual components (Fig. 2).

## Discussion

This study directly compared different anthropometrical and biochemical indicators/measurements as predictors of high blood glucose, hypertension, dyslipidemia and MetS in obese and overweight patients, with a particular focus on WC, WC^*^TG and WC^*^TyG as assessment tools. Our findings revealed that WC^*^TyG index had a significant positive association with MetS and most of the accompanying separate comorbid components. The higher the WC^*^TyG, the greater the prevalence of MetS, which allowed categorizing the population for individualized treatments.

There are not many studies that conjointly compare/discriminate chronic diseases by WC, WC^*^TG and WC^*^TyG, which gives value to the importance of establishing reference points that can be applied to describe the prevalence of some diseases was done in this study. This approach can also be used to identify individuals who may be at risk, or even to determine the type and intensity of treatment, as well as to evaluate its effect in a simple, inexpensive and precise way^8^. Establishing cut-off points for the diagnosis and treatment of some diseases can be very beneficial for personalized nutrition, medical care and to be used at a public health level, as the early detection of these complications will have an impact on healthcare resources^38^.

WC has been used to categorize the type of metabolic risk, as well as to identify individuals with diabetes and cardiovascular disease for early interventions, concerning independent manifestations of the MetS^39,40^. Interestingly, the proposed WC^*^TyG index as a marker of MetS combines anthropometric (WC) and metabolic markers (glucose/TG) that are key variables involved in the definition of MetS according to ATP III, EGIR and IDF criteria^41^.

Indeed, previous studies have indicated that WC is a useful predictor for cardiovascular disease risk factors and coronary heart disease^42,43^. In the current study, significant associations between WC, WC^*^TG and WC^*^TyG index and dyslipidemia on MetS were found. These results are in agreement with other studies that reported a strong association between WC and dyslipidemia with type 2 diabetes mellitus^44^. Furthermore, previous research has demonstrated that WC was positively associated with the prevalence of hypertension^45^. Some authors have warned that the criterion for hypertriglyceridemia has changed from ≥ 150 mg/dL to ≥ 175 mg/dL^46^. Moreover, a recent study has proven that WC^*^TG is a useful marker for the identification of cardiovascular risk^22^. Another trial showed that the TyG index can predict incident hypertension, which supports our findings that triglycerides are linked to hypertension^47^. In this study, the reliability of the TyG index was recognized, due to suitable simplicity and effectiveness. Thus, the existing relationships between this index and chronic diseases, such as diabetes, hypertension, dyslipidemia and MetS, observed in this study complement results of some authors that have previously reported the association of this index with cardiovascular diseases^48^. Furthermore, Jialal et al*.* showed that monocyte and neutrophil ratios together with HDL-c are good predictors of MetS^49^.

In the present study, an association between WC^*^TG and prediabetes, hypertension, dyslipidemia and MetS was detected. To date, most available studies analyzing the association of WC^*^TG with coronary heart disease and cardiovascular disease have considered this marker as a predictive indicator of the occurrence and development of these diseases^50,51^. Thus, Lemieux et al. call this index “the hypertriglyceridemic waist phenotype”, who demonstrated that it was also associated with an increased prevalence of type 2 diabetes in adult men and women, while Fernández-García et al*.* related it to a sedentary lifestyle, which is an important risk factor in the development of obesity-related metabolic diseases^52,53^.

In addition to all these indices, WC^*^TyG index has also been demonstrated as an important index for predicting the risk of high blood pressure, hypertension and MetS. Nonetheless, it should be noted that both the WC^*^TyG and WC^*^TG showed a similar diagnostic performance for MetS. The association of this marker with diabetes and prediabetes was already reported by Zheng et al*.,* in patients with type 2 diabetes mellitus^20^. The novelty of our research focuses on the conjoint examination with other complications such as MetS features, which was positively related because the screening was performed in Caucasians, in whom analyses are scarce.

Interestingly, the WC^*^TyG index to define the hypertriglyceridemic waist phenotype has been successfully compared with the Framingham Score, as well as with other pathological conditions, such as liver and renal disfunction in Australians, prediabetes in Chinese, individual metabolic syndrome components in Spanish, cardiometabolic alterations in Brazilians, diabetes in Mexicans, coronary artery classification in Koreans, incident ischemic stroke in Chinese, incident of hypertension in Iranians. However, there is a need to establish cut-offs/reference points in different populations, as well as to conjointly assess metabolic syndrome features (adiposity, hyperglycemia, hypertension and dyslipidemia) in Caucasians, which is lacking^54,55^. Furthermore, the role of genetics, sex and nutrition can influence the discriminative value of this marker^20,56–58^.

Some authors have evaluated the optimal cut-off points for BMI and WC as a risk factor of certain related diseases in Chinese adults^59^. Other studies have concluded that WC cut-off points represent values for epidemiological identification of risk for hypertension. Furthermore, sensitivity and specificity examinations concerning the cut-off points based on the WC^*^TyG agreed with estimates based on actual measured values^60^. In the current investigation, the results suggest that people over the cut-off points have a statistically higher prevalence of the studied syndromes, which is supported by previously published literature, such as Sabanayagam et al., that established a HbA1C cut-off point to diagnose diabetes^38^.

Several limitations may have concurred in the current analyses. First, the number of participants in the study was relatively small. Second, the population studied included obese people, therefore the data may not be extrapolated to the general population, nonetheless this was a required condition for the MetS phenotype. Third, the low number of diabetes cases may have impacted our results. Instead, we decided to study high blood pressure as an outcome. Due to the characteristics of the population there might be some selection bias. Nevertheless, we should emphasize the novelty of the present investigation in Caucasian subjects, given it is the first study that proposes reference points on combined markers useful for the diagnosis of MetS phenotype and defining components.

Further studies in other populations are needed to replicate these findings, but it appears that the anthropometric measurement of WC at the waist (adiposity) combined with TyG index (lipid/glucose metabolism markers) is a good integrated estimator of MetS, despite age, sex, race and other factors, in order to conjointly discriminate the MetS phenotype with an integrated perspective, where the estimated cut-off (842.7) provided a good AUC value of 0.80 to feature subjects with MetS^61^.

## Conclusion

In clinical settings, WC*TyG is an index that consists of two indicators that are easy to measure (triglycerides and glucose), and interestingly it can serve as an integrated complement of other diagnostic index (as HOMA) for defining clinically complex relevant diseases such as metabolic syndrome. Indeed WC*TyG appears as an index of the hypertriglyceridemic waist phenotype and the proposed reference points are valuable indicators, but not the only ones, to objectively define MetS and clinically associated manifestations.

The greatest novelty of this index is that it is an objective index that integrates various anthropometrical and biochemical factors within a reliable analysis at a quantitative level, which leads to better precision nutrition.

## Supplementary Information

Supplementary Information.

## Abbreviations

ATP III: Adult treatment panel IIII
AUC: Area under the curve
BMI: Body mass index
CI: Confident interval
DBP: Diastolic blood pressure
DEXA: Dual energy X-ray
EGIR: European Group for the Study of Insulin Resistance
FPG: Fasting plasma glucose
HDL-c: High density lipoprotein cholesterol
IDF: International diabetes federation
LDL-c: Low density lipoprotein cholesterol
MetS: Metabolic syndrome
NCDs: Non-communicable diseases
OR: Odds ratio
ROC: Receiver operating characteristics
SBP: Systolic blood pressure
TG: Triglycerides
TyG: Triglyceride glucose index
VAI: Visceral adiposity index
WC: Waist circumference
