# Supplementary material for: The hypertriglyceridemic-waist phenotype as a valuable and integrative mirror of metabolic syndrome traits
Source: Sci Rep. 2021 Nov 8;11:21859. doi: 10.1038/s41598-021-01343-x (PMC8575863; doi:10.1038/s41598-021-01343-x)
Supplement: Supplementary file 1 — Supplementary Information. [file 41598_2021_1343_MOESM1_ESM.docx]

# The hypertriglyceridemic-waist phenotype as a valuable and integrative mirror of metabolic syndrome traits

Begoña de Cuevillas^1^, Ismael Alvarez-Alvarez^1^, Jose I. Riezu-Boj^1,2^, Santiago Navas-Carretero^1,2,3^ and J. Alfredo Martínez^1,2,3,4^

^1^Department of Nutrition, Food Sciences and Physiology, Center for Nutrition Research, University of Navarra, 31008 Pamplona, Spain.

^2^IdisNA Health Research Institute of Navarra, Pamplona, Spain.

^3^CIBER Physiopathology of Obesity and Nutrition (CIBEROBN), Institute of Health Carlos III, 28029 Madrid, Spain.

^4^Precision Nutrition Program, Cardiometabolic IMDEA Food, 28049 Madrid, Spain.

Correspondence: Santiago Navas-Carretero

Department of Nutrition, Food Science, and Physiology. Center of Nutrition Research. Universidad de Navarra. Calle Irunlarrea 1, 31008, Pamplona.

Tel +34 948425600 Ext. 80-6623

Fax +34 948 42 56 49

Email : [snavas@unav.es](mailto:snavas@unav.es)

**Supplemental table 1.** Diagnostic performance of the estimated cut-off points to detect metabolic diseases by the different indices.

| **Index** | **AUC** | **95% CI** | **p-value** | **Sensitivity at optimal cut-off point** | **Specificity at optimal cut-off point** | **Cut-off** |
| --- | --- | --- | --- | --- | --- | --- |
| **High blood glucose** |  |  |  |  |  |  |
| Waist circumference | 0.65 | 0.58, 0.72 | ***<0.001*** | 0.68 | 0.57 | 102.6 |
| Waist circumference^*^TG | 0.70 | 0.63, 0.77 | ***<0.001*** | 0.64 | 0.69 | 116.0 |
| Waist circumference^*^TyG | 0.73 | 0.67, 0.80 | ***<0.001*** | 0.65 | 0.73 | 904.7 |
| **Hypertension** |  |  |  |  |  |  |
| Waist circumference | 0.71 | 0.65, 0.78 | ***<0.001*** | 0.62 | 0.72 | 93.7 |
| Waist circumference^*^TG | 0.71 | 0.65, 0.77 | ***<0.001*** | 0.65 | 0.67 | 101.5 |
| Waist circumference^*^TyG | 0.75 | 0.69, 0.80 | ***<0.001*** | 0.83 | 0.53 | 794.6 |
| **Dyslipidemia** |  |  |  |  |  |  |
| Waist circumference | 0.67 | 0.61, 0.74 | ***<0.001*** | 0.70 | 0.58 | 97.0 |
| Waist circumference^*^TG | 0.83 | 0.78, 0.89 | ***<0.001*** | 0.68 | 0.86 | 135.7 |
| Waist circumference^*^TyG | 0.77 | 0.69, 0.80 | ***<0.001*** | 0.60 | 0.81 | 918.8 |
| **Metabolic syndrome** |  |  |  |  |  |  |
| Waist circumference | 0.72 | 0.66, 0.78 | ***<0.001*** | 0.76 | 0.62 | 102.3 |
| Waist circumference^*^TG | 0.81 | 0.75, 0.86 | ***<0.001*** | 0.66 | 0.85 | 135.7 |
| Waist circumference^*^TyG | 0.81 | 0.76, 0.87 | ***<0.001*** | 0.69 | 0.83 | 915.3 |

Waist circumference cut-off points are shown in cm. Waist circumference^*^TG and Waist circumference^*^TyG cut-off points are shown in cm^*^mg/dl.

**Supplemental table 2.** Sex-specific associations between indices (below or above the specific cut-off point) and metabolic syndrome and its components.

| **MEN** | | | | | | | | |
| --- | --- | --- | --- | --- | --- | --- | --- | --- |
| **Index** | **Cut-off** | **High blood glucose** | **Cut-off** | **Hypertension** | **Cut-off** | **Dyslipidemia** | **Cut-off** | **Metabolic syndrome** |
| Waist circumference | Low (<101.6) | 1 (reference) | Low (<116.1) | 1 (reference) | Low (<105.1) | 1 (reference) | Low (<105.1) | 1 (reference) |
|  | High (≥101.6) | 1.71 (0.40-7.20) | High (≥116.1) | -^‡^ | High (≥105.1) | 3.64 (1.23-10.79) | High (≥105.1) | 3.12 (1.10-8.83)^*^ |
| Waist circumference^*^TG | Low (<135.8) | 1 (reference) | Low (<183.4) | 1 (reference) | Low (<121.5) | 1 (reference) | Low (<121.5) | 1 (reference) |
|  | High (≥135.8) | 3.37 (1.09-10.40)^*^ | High (≥183.4) | 6.43 (0.65-63.91)^*^ | High (≥121.5) | 18.02 (4.61-70.48)^*^ | High (≥121.5) | 7.81 (2.52-24.25)^*^ |
| Waist circumference^*^TyG | Low (<926.2) | 1 (reference) | Low (<915.4) | 1 (reference) | Low (<884.0) | 1 (reference) | Low (<898.8) | 1 (reference) |
|  | High (≥926.2) | 3.36 (1.07-10.59)^*^ | High (≥915.4) | 2.80 (0.76-10.33)^*^ | High (≥884.0) | 30.761 (3.8-251.0)^*^ | High (≥898.8) | 14.90 (3.62-61.27)^*^ |
|  | | | | | | | | |
| **WOMEN** | | | | | | | | |
| **Index** | **Cut-off** | **High blood glucose** | **Cut-off** | **Hypertension** | **Cut-off** | **Dyslipidemia** | **Cut-off** | **Metabolic syndrome** |
| Waist circumference | Low (<102.6) | 1 (reference) | Low (<102.3) | 1 (reference) | Low (<101.2) | 1 (reference) | Low (<92.6) | 1 (reference) |
|  | High (≥102.6) | 1.89 (0.89-4.01) | High (≥102.3) | 3.16 (1.68-5.93)^*^ | High (≥101.2) | 2.39 (1.20-4.76) | High (≥92.6) | 3.34 (1.20-9.33)^*^ |
| Waist circumference^*^TG | Low (<116.0) | 1 (reference) | Low (<84.8) | 1 (reference) | Low (<73.4) | 1 (reference) | Low (<84.8) | 1 (reference) |
|  | High (≥116.0) | 2.93 (1.37-6.24)^*^ | High (≥84.8) | 3.20 (1.73-5.92)^*^ | High (≥73.4) | 7.07 (2.55-19.63)^*^ | High (≥84.8) | 4.63 (1.92-11.18)^*^ |
| Waist circumference^*^TyG | Low (<905.2) | 1 (reference) | Low (<778.3) | 1 (reference) | Low (<748.9) | 1 (reference) | Low (<823.6) | 1 (reference) |
|  | High (≥905.2) | 3.91 (1.78-8.59)^*^ | High (≥778.3) | 4.07 (2.09-7.94)^*^ | High (≥748.9) | -^¥^ | High (≥823.6) | 3.49 (1.63-7.49)^*^ |

^*^ p<0.05

^‡^ Waist circumference above the cut-off point predicts the outcome perfectly.

^¥^ Waist circumference^*^TyG above the cut-off point predicts the outcome perfectly.

Adjusted for age, physical activity, family history of obesity, energy intake, adherence to a Mediterranean dietary pattern and education level.
